# No Geographic Distribution Change Among Residency Applicants in the Neurology Match During COVID-19

**DOI:** 10.7759/cureus.34898

**Published:** 2023-02-12

**Authors:** Paul Beinhoff, Nabil Attlassy, Chad Carlson

**Affiliations:** 1 Neurology, Medical College of Wisconsin, Milwaukee, USA

**Keywords:** geographic bias, residency match, neurology, virtual interview, medical education, covid-19

## Abstract

Objectives

The COVID-19 pandemic posed a novel challenge for the 2020-2021 Match cycle resulting in a virtual interview season. The advent of virtual interviews raised concerns for both programs and medical students. The possibility of an impact on the application strategies for medical students resulting in students being more likely to remain in the region or state of their medical school was considered. We investigated whether there was a change in the geographic distribution of residency applicants for the class of 2025 (matched in 2021) as compared to the previous three application cycles (classes of 2022-2024) across all 168 neurology residency programs within the United States.

Methods

Publicly available data from neurology program websites were compiled to record the location of resident medical schools and matched programs for the residency classes of 2022-2025. Missing or ambiguous information was cross-referenced to social media, (e.g. LinkedIn and Twitter). Statistical analyses were conducted utilizing SPSS 26 (IBM SPSS 26 Statistics for Windows, Armonk, NY).

Results

Across all four classes, regional retention (students matching in the same region) was 70.2% for the Northeast, 59.6% for the Midwest, 52.9% for the South, and 59.4% for the West. No significant change between the residency class of 2025 and the previous three classes was present.

Discussion

No significant change to the geographic trends for candidates was seen with the virtual interview process for the 2020-2021 neurology Match. As has been seen in other fields, a strong regional preference, with the majority of residents matching to programs in the same regions as their medical school, was seen for neurology.

## Introduction

The COVID-19 pandemic has challenged healthcare and medical education in numerous ways. In 2020, two major changes occurred with the potential to impact the residency Match process. The first was the AAMC (Association of American Medical Colleges) announcement that audition/away rotations were discouraged, except under limited circumstances, and were restricted to one per applicant [1]. Audition rotations are often popular among fourth-year medical students to introduce themselves to programs and secure letters of recommendation. Within some specialties, audition rotations are a key part of the application and interview process [2]. Across all specialties, 54.7% of 15,860 survey respondent senior medical students in 2019 completed at least one audition rotation [3]. The second change was the transition to a virtual interview process for the 2020-2021 residency and fellowship Match cycle.

Geography has been an important factor for applicants when selecting where to apply and match for residency [4-6]. A nationwide review of NRMP (National Resident Matching Program) data for US allopathic seniors from 2011-2015 found that 63% matched to programs within the same region as their medical school [4]. This “home field” preference has been reported for plastic surgery, otolaryngology, orthopedic surgery, and general surgery [7-12]. A similar impact of geographic preferences on program rank-list choices has been reported [13]. The abrupt transition to virtual interviews in the setting of a global pandemic raised the concern that geography could become an even greater factor. This concern was supported by data from plastic surgery showing an increase in the number of students matching to their home program or programs within the same region (particularly in the Midwest) [14,15].

In this study, we explored the impact of the virtual interview cycle on the neurology Match. Our hypothesis was that an increase in the “home field” preference would be seen, leading to more candidates matching within the same state or region than in previous years. As a secondary aim, we wanted to explore the impact of training type (allopathic versus osteopathic) on regional matching trends. Our secondary aim was to investigate in order to better understand the relationship between geographic distribution and applicant characteristics.

## Materials and methods

Our findings were exempt from institutional review board review as only publicly available information was utilized (secondary use of information exemption). A list of all the Accreditation Council for Graduate Medical Education (ACGME) accredited neurology residency programs were compiled using the AMA’s (American Medical Association) residency and fellowship electronic interactive database (FREIDA). Publicly available data from program websites and social media were abstracted. When residency class years weren’t specified, resident LinkedIn and Twitter profiles were located to cross-check and confirm data on the website. Programs were excluded if they did not have publicly available lists of current residents with data indicating their medical school or if data on a resident’s current year in training could not be verified through the program website or social media. Advanced programs, programs that match residents only for the three primary years of neurology and not the initial intern year, were excluded as neurology residents from the virtual interview season would not have been enrolled in a neurology residency at the time of data abstraction. Similarly, categorical programs which did not include data for their PGY1 (Post-Graduate Year) class were excluded; the data had to be complete for the entire four years.

For included programs, each resident from the class of 2025 (current interns) to the class of 2022 (current PGY4s) was included. Abstracted data included planned graduation year, medical degree (e.g. MD, DO), medical school, medical school state, medical school region, residency program, residency program state, residency program region, and the date the data were abstracted. We classified medical degrees into three categories: MD, DO, and other equivalents (e.g. MBChB and MBBS).

Four regions were designated based on the United States Census Bureau statistical regions [16]. Programs in Washington D.C. were considered part of the “South” region and Washington D.C. was considered as its own “state” for “in-state” purposes. Graduates from medical schools outside of the United States were defined as international for state and regional designations, including Puerto Rico. If an applicant’s medical school and residency program were located in the same state, this was defined as “in-state” as well as “in-region”. Applicants attending a residency within the same region, but different state, were defined only as “in-region.”

Data were analyzed utilizing SPSS 26 (IBM SPSS 26 Statistics for Windows, Armonk, NY). Categorical comparisons were analyzed utilizing chi-square tests. Residents that graduated from medical schools outside of the United States were excluded from the analysis, as, by definition, they could not have a preference for their local region when applying to residency programs within the United States.

## Results

Data were abstracted between August 30th, 2021 and October 23rd, 2021. Of the 168 programs, 75 (44.6%) met our inclusion criteria. Residents from the 93 programs that did not meet the inclusion criteria were excluded; the reasons for exclusion are summarized in Table 1 and the type of programs (sorted by region) that were excluded are shown in Table 2. Programs were included from all four regions: 24/39 (61.5%) for the Midwest, 10/29 (34.5%) for the West, 18/43 (41.9%) for the Northeast, 23/57 (40.4%) for the South.

**Table 1:** Criteria for Exclusion Within each of the four regions for all residency classes, the number of programs excluded for each of these criteria was listed. Advanced programs Match only of PGY2-4 (Post-Graduate Year) residents. Previous institutions not listed refer to absent medical school listings.

	Midwest (15)	West (19)	Northeast (25)	South (34)
Previous institutions not listed	10	3	3	12
Advanced program	3	5	15	7
No residents information listed	1	9	3	10
Resident data not up-to-date	1	1	1	3
No senior resident data included	0	1	3	2

**Table 2:** Program Type for Excluded Programs The number of programs for each region that were excluded are sorted based upon the type of program for all residency classes. Program type was classified by FREIDA designation. FREIDA: Fellowship and Residency Electronic Interactive Database Access

	Midwest (15)	West (19)	Northeast (25)	South (34)
University-based	9	10	15	21
Community-based university-affiliated	4	4	8	7
Community based	2	4	2	4
Military based	0	1	0	2
Total percent excluded by region	35.9%	65.5%	58.1%	59.6%

Virtual versus in-person interviews and in-state or in-region matches

**Figure 1:**
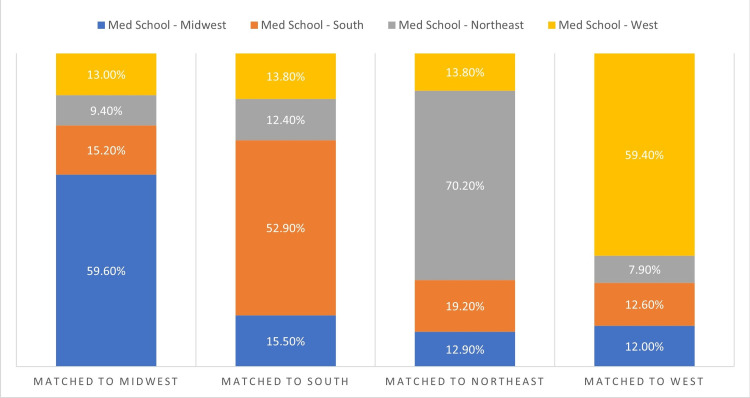
Regional Distribution of Matched Residents The distribution of matched residents within each of the four regions is shown for all residency classes. Within each region, results are broken down based upon the region in which the resident’s medical school was located.

Table 3 summarizes the number of students matching from each state as well as the number of residents matched within each state. Graduates of medical schools outside of the United States comprised 26.1% of residents. Of the 1,433 current neurology residents that graduated from medical schools in the United States, 439 (30.6%) remained in the same state for residency and 868 (60.6%) remained within the same region. In all four regions, a majority of medical school graduates matched to programs in the same region: 59.6% for the Midwest, 52.9% for the South, 70.2% for the Northeast, and 59.4% for the West (Figure 1).

**Table 3:** Distribution of Medical School Applicants and Residency Programs by State for all Residency Classes Each individual applicant was sorted by the state in which their medical school was located and the state in which their matched residency program was located. Graduates from medical schools outside of the US are included in the total matched residents per state.

	Medical School	Residency Match		Medical School	Residency Match
Alabama	30	35	Oregon	4	19
Arizona	16	39	Pennsylvania	143	141
Arkansas	5	16	Rhode Island	8	24
California	66	124	South Carolina	9	19
Colorado	20	33	South Dakota	7	0
Connecticut	18	40	Tennessee	29	28
DC	21	44	Texas	86	109
Florida	58	64	Utah	8	30
Georgia	31	59	Virginia	32	23
Hawaii	4	0	Washington	12	0
Illinois	88	128	West Virginia	8	0
Indiana	65	31	Wisconsin	22	22
International	508	0	Vermont	3	16
Iowa	18	31	Nebraska	17	27
Kansas	14	0	Nevada	7	8
Kentucky	18	27	New Hampshire	5	0
Louisiana	25	30	New Jersey	22	20
Maine	4	0	New Mexico	3	0
Maryland	21	26	New York	152	222
Massachusetts	47	107	North Carolina	32	55
Michigan	93	141	North Dakota	5	0
Minnesota	19	56	Ohio	62	42
Mississippi	10	0	Oklahoma	14	0
Missouri	52	105	Total	1941	1941

For candidates interviewed in traditional interview years (classes of 2022-2024), 30.6% matched to programs in the same state compared with 30.9% of those that had virtual interviews (class of 2025) during the COVID-19 pandemic (p=0.948). No difference was seen between traditional and virtual interviews at the regional level (p=0.668) with 60.9% of traditional and 59.6% of virtual candidates remaining in the same region. The percentage of students matching to a program within the same region is stratified by year as well as by virtual versus traditional interview years in Table 4. No differences were seen between individual years nor between the two interview types for all four regions.

**Table 4:** Regional Variance by Class Year (Year of Planned Graduation From Residency) and Traditional Versus Virtual Years Chi-square analyses were conducted across all four years for each region, listed within the “between years” column. A Fisher’s exact test was utilized to assess for differences within the “traditional vs virtual” column.

	Class of 2022	Class of 2023	Class of 2024	Class of 2025	Between Years	Traditional (Class of 2022-2024)	Virtual (Class of 2025)	Traditional vs Virtual
Midwest	65%	62%	49%	62%	p=0.083	59%	62%	p=0.593
South	50%	62%	49%	51%	p=0.24	53%	51%	p=0.739
East	79%	71%	66%	65%	p=0.096	72%	65%	p=0.342
West	62%	51%	67%	61%	p=0.604	59%	61%	p=0.846

Distribution of international, osteopathic, and allopathic graduates

Among the included programs, the regional distribution of international medical graduates (IMGs) shows a small plurality (36%) matching within the Midwest (Table 5). The percentage of available positions within the included programs matching IMGs ranges from 11-32% (Table 5).

**Table 5:** Regional Distribution of International Medical Graduates Results are broken down based on the percentage of IMGs within a region and the percentage of IMGs among all available positions for all residency classes. IMG: international medical graduate

	% of IMGs in the region	% of total positions filled by IMGs in the region
Midwest	36%	32%
South	33%	31%
East	25%	22%
West	6%	11%

**Figure 2:**
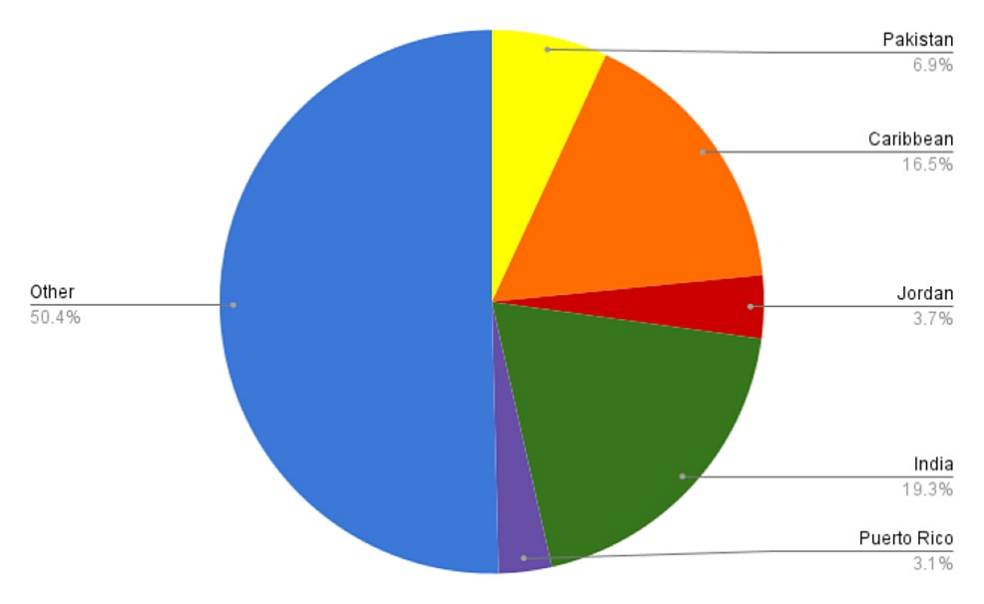
Medical School Country of Origin for International Medical Graduates The five most prevalent countries of origin for IMG’s meeting inclusion criteria are shown in pie chart form.

**Figure 3:**
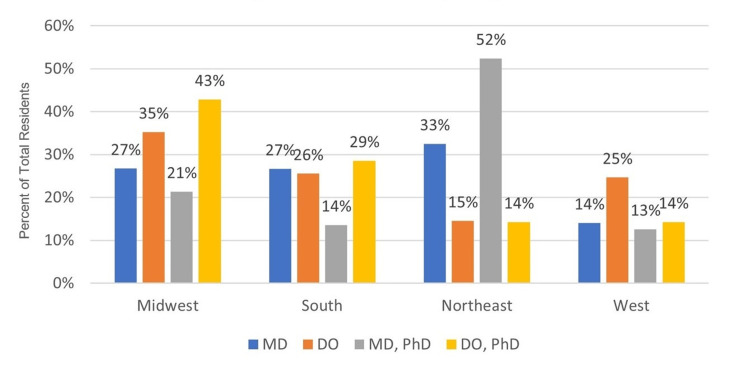
Regional Variance in the Degree Type of Matched Residents from US Medical Schools The distribution of Matched residents, broken down by the degree type, within each of the four regions is shown among all residency classes. MD: Doctor of Medicine; DO: Doctor of Osteopathic Medicine; PhD: Doctor of Philosophy

The regional variation of degree types (osteopathic versus allopathic including combined programs) demonstrates a statistically significant difference (p<0.001) across all groups with the plurality of MD and the majority of combined MD and PhD program graduates matching within Northeast programs whereas the plurality of DO and combined DO and PhD program graduates matched to Midwest programs (Figure 3). Graduates from allopathic or osteopathic programs did not have significant differences in their rates of remaining in the same state (p=0.246) or the same region (p=1) as their medical school.

## Discussion

This retrospective analysis of the impact of virtual interviews during the COVID-19 pandemic is the first to assess the geographic trends for neurology applicants and the impact of virtual interviews during the pandemic. We found that a majority (60.6%) of applicants in neurology matched to programs in the same geographic region and 30.6% matched to programs in the same state as their medical school. There was no difference in the percentage of graduates staying in the same region between traditional interview years (60.9%) versus the class with a virtual interview during the pandemic (59.6%).

Evaluating the possibility of unexpected impacts of the virtual interview process for the 2020-2021 application cycle was the primary focus of this study. In the integrated plastic surgery Match, an increase in the percentage of residents matching in the same region [14,15] and an increase in programs matching internal candidates [15] was observed. This is in contrast to neurology, where no significant changes in the national or regional matching patterns were identified. Across the four years for which data were abstractable, the Midwest retained 49-65% of applicants from the region, the South retained 49-62% of applicants from the region, the Northeast retained 65-79% of applicants from the region, and the West retained 51-67% of applicants from the region (see Table 4). The stability of the final results compared across years is reassuring, however, it does not rule out other potential impacts of virtual interviews. Potential factors that cannot be evaluated with this retrospective data set include the geographic distribution of interview offers from programs and final program rank list preferences; previous studies have demonstrated geographic preferences in program rank lists [13]. It is possible that the virtual interview process leads programs to favor regional candidates for interview positions (e.g. “known commodities”), however, the lack of difference between virtual and traditional interview years suggests that any such change is likely modest.

The significance of no observable change in geographic distribution for neurology lies in supporting virtual interviews as a viable alternative to in-person interviews for future application cycles. These data support no significant change to the Match with regard to geographic program preferences when comparing between virtual and traditional Match cycles. While same-institution matches were not analyzed, we confirmed a “home field advantage” at the regional level similar to what has been previously reported [4]. This may be influenced by applicant behaviors and more recently the supplemental application. With the new supplemental application, applicants will have the option to submit additional experience information, geographic preferences, and up to three individual program signals. While ensuring the stability of the Match process is critical, other factors may play a role in future decisions to extend virtual or in-person interviews. Programs may consider continuing virtual interviews due to the reductions in both cost and administrative burden [17]. However, applicants tend to favor in-person interviews as more capable of communicating program culture and socio-environmental factors [17, 18].

The changes to away (audition) rotations may have been a factor in the “home field” preference differences that were seen in plastic surgery, but not in neurology [14, 15]. In plastic surgery during the 2020 residency application cycle, 97.4% of applicants reported completion of at least one or more away rotations as compared to 39.3% for neurology applicants [3]. Among the students that completed an away rotation, the median number of away rotations for neurology residency applicants was one compared to three for plastic surgery in 2020 [3]. Away rotations are generally considered of greater value in the decision-making process for programs in plastic surgery compared to neurology. Away rotations in one study were described as the most valuable application component for plastic surgery criterion in selection by program directors [2] whereas away rotations were ranked among the least important factors in selecting applicants for neurology [19]. While away rotation changes may have impacted application and interview strategies for both candidates and programs, no significant changes to the geographic distribution were seen for neurology.

As a secondary aim of this investigation, we assessed regional variation between allopathic, osteopathic, and international graduates. The plurality of osteopathic (35%) and international (36%) graduates matched to programs in the Midwest; for candidates with dual degrees, the majority of MD and PhD graduates matched to programs in the Northeast whereas a plurality of DO and PhD graduates matched to the Midwest. There were no significant changes in DO regional distributions. IMG regional distributions were inherently not significantly changed per US graduate geographic distribution not significantly changing during the virtual interview process.

The retrospective nature of the data gathered and the lack of reporting from many programs are limitations of this study. Advanced program data also were not available as those matched applicants will be enrolled in intern years which may or may not be in the institution at which they will complete neurology residency, thus further limiting our total sample size. As a result, only 44.6% of neurology programs had analyzable data. Nonetheless, we were able to analyze data for 1,433 residents who graduated from schools in the United States and were currently enrolled in neurology programs across the four regions of the United States. While all regions were represented, there was regional variation in the number of programs with complete data ranging from 61.5% for the Midwest down to 34.5% for the West. In the future, we hope that fewer programs will have incomplete data, allowing for future analyses to evaluate the stability of geographic matching trends in our field and also allowing applicants to have a more complete picture of all programs [20]. We were also unable to assess resident-specific or program-specific factors that may lead to a bias toward a particular school or region. A future project involving direct survey data from residents and program directors could clarify factors beyond the modality of interview methods capable of impacting regional trends.

## Conclusions

This study demonstrates no observable impact of virtual interviews during the COVID-19 pandemic on the geographic distribution of matched residents in neurology. Furthermore, it confirms the “home field” preference for neurology programs in the same region as an applicant’s medical school. These findings do suggest an important role for geographic signaling in the Supplemental Application for candidates preferring to relocate to regions distant from their medical school; ultimately the impact of geographic and preference signaling remains to be seen for neurology. As graduate medical education debates the relative merits of in-person versus virtual interviews, our findings are reassuring that the two interview types produce similar geographic distributions of residents. However, further research is warranted to understand other potential impacts (both beneficial and detrimental) of virtual interviews for both candidates and programs.
